# Prognostic and immunological potential of PPM1G in hepatocellular carcinoma

**DOI:** 10.18632/aging.202964

**Published:** 2021-05-05

**Authors:** Yi-Ren Lin, Wen-Jing Yang, Guo-Wang Yang

**Affiliations:** 1 Department of Oncology, Shunyi Hospital of Beijing Traditional Chinese Medicine Hospital, Beijing, China; 2 Department of Oncology, Beijing Hospital of Traditional Chinese Medicine, Capital Medical University, Dongcheng, Beijing, China

**Keywords:** PPM1G, hepatocellular carcinoma, prognosis, spliceosome, immune infiltration

## Abstract

Liver hepatocellular carcinoma (LIHC) remains one of the most common causes of cancer death. Prior research suggested that the PPM1G gene is involved in LIHC. To explore the role of PPM1G in LIHC, we used several online databases. Expression profiling was performed via the Gene Expression Profiling Interactive Analysis (GEPIA), Hepatocellular Carcinoma Database (HCCDB), Oncomine and Human Protein Atlas (HPA) platforms. Mutation profiles were investigated via cBio Cancer Genomics Portal (cBioPortal). Survival analysis was performed via the Kaplan–Meier (KM) plotter and International Cancer Genome Consortium (ICGC) platforms. The biological function of PPM1G was analyzed via the Enrichr database. The influence of PPM1G expression in the tumor immune microenvironment was assessed via Tumor Immune Estimation Resource (TIMER). PPM1G expression was upregulated in various tumors, including LIHC. Overexpression of PPM1G was associated with poor prognosis in LIHC. PPM1G expression might be regulated by promoter methylation, copy number variations (CNVs) and kinases and correlate with immune infiltration. The gene ontology (GO) terms associated with high PPM1G expression were mRNA splicing and the cell cycle. The results suggest that PPM1G is correlated with the prognosis of LIHC patients and associated with the tumor immune microenvironment in LIHC.

## INTRODUCTION

Cancer remains a considerable threat to human survival. The Global Burden of Disease Study reported that there were 24.5 million cases of cancer worldwide and 9.6 million cancer deaths in 2017 [1]. Liver hepatocellular carcinoma (LIHC) is one of the most common causes of cancer death. In 2017, there were 953,000 (95% UI, 917,000-997,000) incident cases of liver cancer globally and 819,000 (95% UI, 790,000-856,000) deaths [1]. Unlike many solid tumors, the incidence and mortality of LIHC have increased over the past decade [2]. Many LIHC patients still have a poor survival prognosis after surgery, radiotherapy or chemotherapy owing to late diagnosis. Therefore, it is of great urgency to conduct more research on LIHC.

Epidemiological research has shown that more than 80% of hepatocellular carcinomas develop in fibrotic or cirrhotic livers [3]. PPM1G plays a fundamental role in increasing liver fibrosis by negatively regulating the effect of WWP2 on Notch3 degradation [4]. The inhibition of PPM1G activity by CdCl2 also decreases the protein levels of fibrogenic markers [5]. It is tempting to speculate that the biological function of PPM1G is associated with LIHC.

PPM1G is a member of the metal-dependent protein phosphatase (PPM) family. Previous studies demonstrated that malfunction of the PPM family was correlated with tumors, metabolic diseases and other various diseases. Protein phosphatases control diverse cellular events, including proliferation, differentiation, and stress response, by regulating reversible protein phosphorylation (the most important posttranslational modification) [6]. PPM1D, a member of the PP2C family that is recognized as a common oncogene, is related to many different human tumors, including adult supratentorial diffuse astrocytoma and oligodendroglioma, high-grade glioma [7], non-small-cell lung cancer [8] and lymph node metastasis, as well as esophageal squamous cell carcinoma (ESCC) [9]. Some research has indicated that PPM1D is a potential biomarker for prostate cancer, gastric cancer and colorectal cancer [10].

PPM family members, also known as protein phosphatase 2C (PP2C) phosphatases, are Ser/Thr phosphatases that bind manganese/magnesium ions (Mn2+/Mg2+) in their active center and act as a single subunit enzyme. PPM phosphatases are involved in regulating various cell functions, including cell cycle control, cell differentiation, the immune response and cellular metabolism, in twenty different mammals. Mutation, overexpression or deletion of PPM phosphatase genes can cause abnormal cellular responses, leading to various human diseases, including cancer. Therefore, members of the PPM family are considered potential targets for cancer therapy [6].

PPM1G can dephosphorylate pre-mRNA splicing factors, which are essential for the formation of functional spliceosomes. Pre-mRNA splicing is important in the pathology of numerous diseases, especially cancers, because it affects protein diversity [11]. The connection between cancer biology and splicing regulation is of primary importance for understanding the mechanisms leading to disease and improving the development of therapeutic approaches [12]. Therefore, dysfunction of PPM1G may induce cancer progression by affecting pre-mRNA splicing.

In addition to pre-mRNA splicing, PPM1G is related to chromatin remodeling, DNA damage [13], neurodevelopment [14], Parkinson's disease [15] and alcohol use disorders [16]. However, the biological function of PPM1G in LIHC remains to be identified.

Here, we explored the expression, mutation and relationship with clinical outcomes of PPM1G in LIHC patients through various public online platforms. Then, we revealed regulatory factors and functional networks of PPM1G in LIHC and investigated its role in tumor immunity. Our research provides evidence from multiple perspectives supporting the biofunction of PPM1G in LIHC.

## RESULTS

### PPM1G expression in various cancers

**Figure 1:**
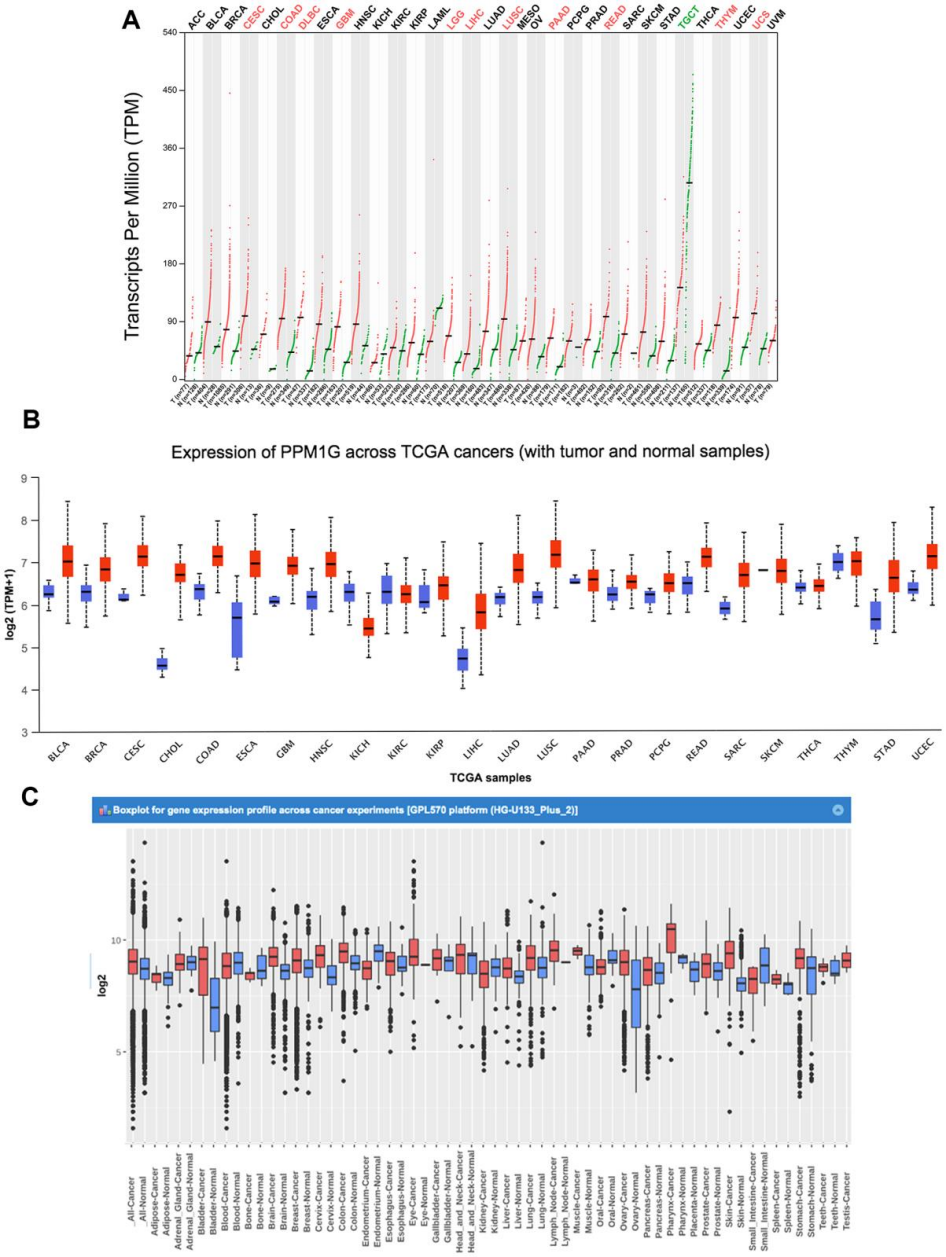
**PPM1G mRNA expression in various types of cancer.** (**A**) The expression of PPM1G in 33 types of human cancer (GEPIA). The gene expression profiles across all tumor samples and paired normal tissues are shown in a dot plot, and each dot represents the expression profile in one sample. (**B**) PPM1G expression in 24 types of cancers (UALCAN). The box plot shows the gene expression levels in different cancers and normal tissues as the interquartile range (IQR), including the minimum, 25th percentile, median, 75th percentile and maximum values. Red boxes represent tumor tissues, and green boxes represent normal tissues. (**C**) Data concerning PPM1G mRNA expression in various types of cancer (GENT). The boxes represent the median and the 25th and 75th percentiles. The dots represent outliers. The red boxes represent tumor tissues, and the green boxes represent normal tissues.

We analyzed the PPM1G expression patterns in the results of various cancer studies from the data of The Cancer Genome Atlas (TCGA) and Gene Expression Omnibus (GEO). Gene Expression Profiling Interactive Analysis (GEPIA) (which uses TCGA data) showed that compared with that in normal tissues, the PPM1G mRNA levels were significantly higher in 11 types of cancer tissues (Figure 1A). The UALCAN website (which uses Liver Hepatocellular Carcinoma Project of The Cancer Genome Atlas (TCGA-LIHC) data) also showed that the PPM1G expression level in tumor tissues was significantly higher than that in normal tissues (Figure 1B). In the Gene Expression across Normal and Tumor tissue (GENT) database (which uses GEO data), whose resources were based on GPL570 clinical data from 29 cancer types of GEO database, which was different from GEPIA and UALCAN database, PPM1G expression levels were found to be upregulated in various cancers, including bladder cancer, breast cancer, colon cancer, lung cancer, pancreatic cancer, liver cancer, ovarian cancer, and testicular cancer (Figure 1C). In the above three online databases, the mRNA expression of PPM1G in LIHC tissues was significantly higher than that in adjacent normal tissues. The results above demonstrated that PPM1G transcription was significantly increased in multiple cancers as well as in LIHC.

### PPM1G expression in LIHC

**Figure 2:**
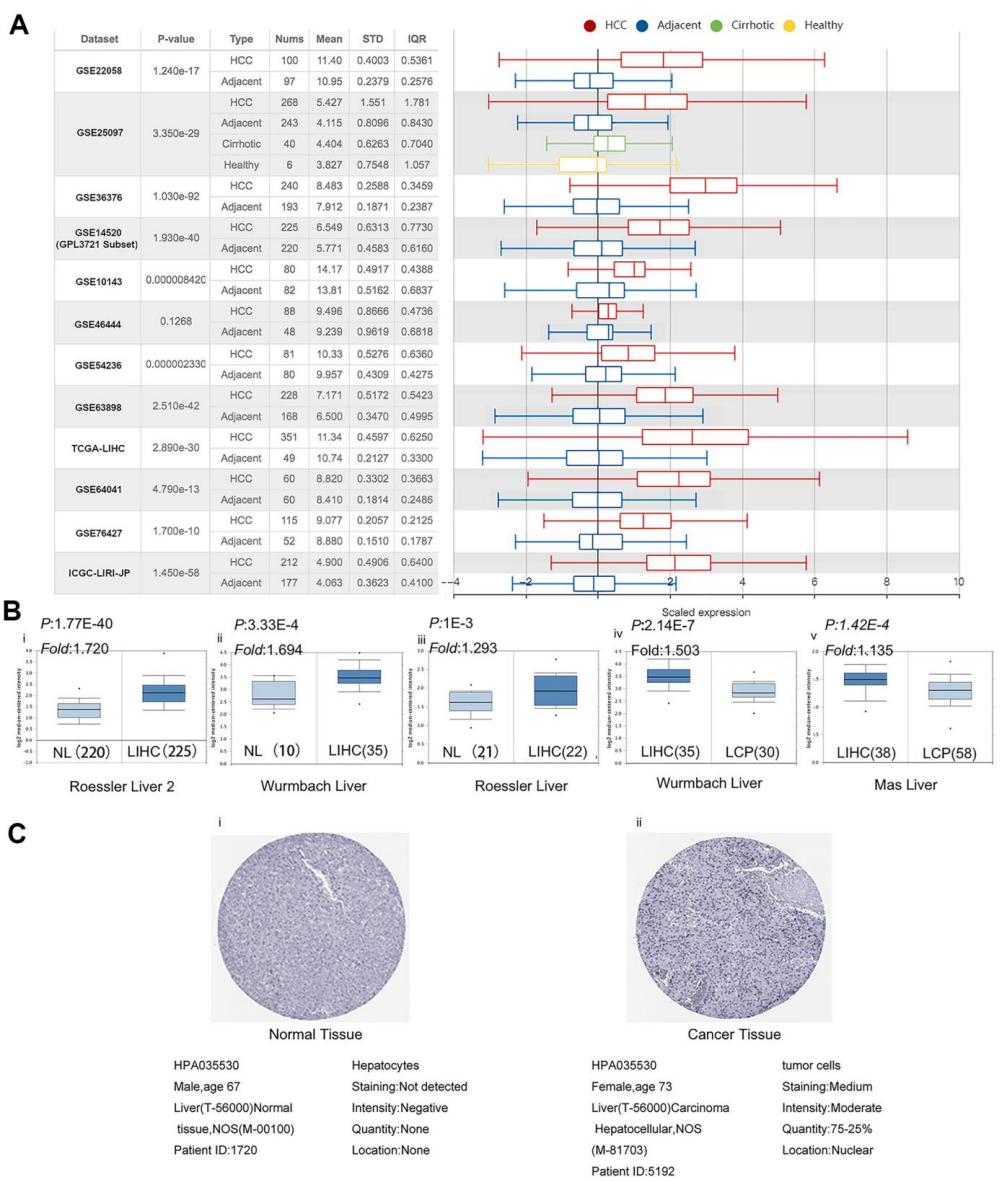
**PPM1G expression is significantly upregulated in LIHC.** (**A**) Chart and box plot showing PPM1G expression in normal and LIHC tissues (HCCDB). (**B**) Box plot comparing PPM1G mRNA expression in normal (left plot) and cancer tissues (right plot) generated using Roessier Liver2 (i), Wurmbach Liver (ii), and Roessier Liver2 (iii) data (Oncomine). Box plots comparing PPM1G mRNA expression in cancer tissues (left plot) and liver cancer precursor tissues (right plot) using the Wurmbach Liver (iv) and Mas Liver (v) datasets, respectively (Oncomine) (p-value of 1E−4, fold-change of 2, and gene ranking of 10%). (**C**) PPM1G protein expression in normal (i) and LIHC tissues (ii) (HPA).

Next, we investigated the PPM1G expression levels in 12 LIHC cohorts from TCGA, ICGC, and GEO via HCCDB. The PPM1G mRNA expression in patients with liver cancer was significantly higher than that in normal controls in the following datasets: GSE22058 [17], GSE25097 [18], GSE36376 [19], GSE14520 (GPL3721 subset) [20], GSE10143 [21], GSE54236 [22], GSE63898 [23], GSE64041 [24], GSE76427 [25] (which from GEO database), TCGA-LIHC(which from TCGA data), and Liver Cancer-RIKEN, JP Project from International Cancer Genome Consortium (ICGC-LIRI-JP) (which from ICGC database) (Figure 2A). The data from GEO, ICGC and TCGA databases are independent separately. Next, the mRNA expression patterns of PPM1G in LIHC were further verified by Oncomine, which was different from GEO, TCGA and ICGC database. Data in the Oncomine database showed that PPM1G was overexpressed in LIHC tissues compared with normal tissues in the Roessler Liver, Roessler Liver2, and Wurmbach Liver datasets. Wurmbach Liver and Was Liver data indicated that PPM1G transcription was significantly higher in the LIHC precursor samples than in normal controls (Figure 2B). In Human Protein Atlas (HPA), 5 of the 9 patient’s LIHC tissues stained by HPA035530 had moderate or weak staining, and 2 had strong staining, while all the normal adjacent liver tissue samples showed no staining (Figure 2C). The HPA images indicated that the protein expression levels of PPM1G were significantly higher in LIHC tissues than in normal controls. The above data showed that the mRNA and protein expression of PPM1G was significantly higher in LIHC tissues than in normal tissues.

### Correlation of PPM1G expression with clinicopathological characteristics of LIHC patients

**Figure 3:**
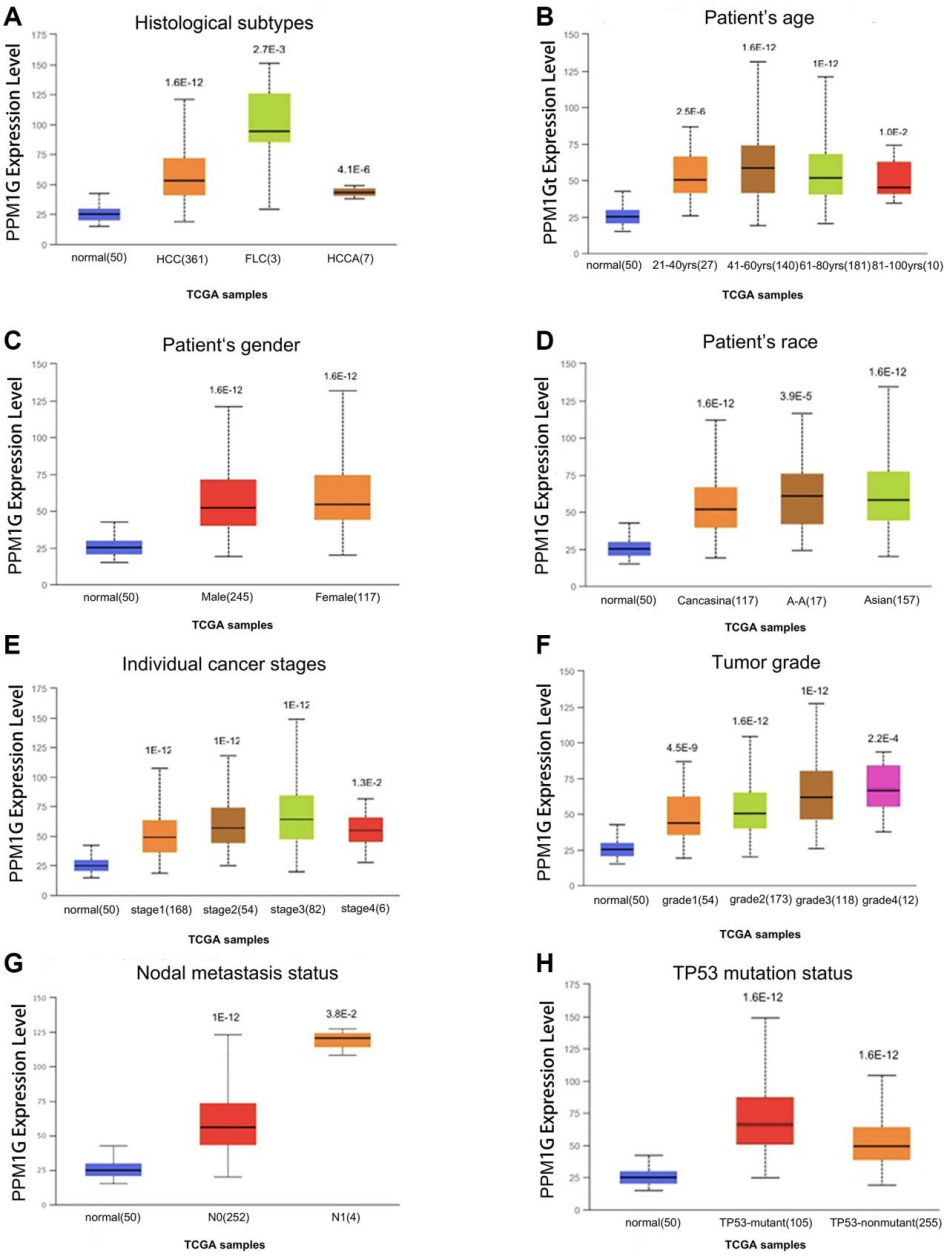
**PPM1G transcription level in subgroups of patients with LIHC stratified based on sex, age and other criteria (UALCAN).** Box-whisker plots showing PPM1G expression in LIHC (different color plots) and normal (blue plots) tissues in patient subgroups based on (**A**) histological subtype, (**B**) patient age, (**C**) patient sex, (**D**) patient race, (**E**) individual cancer stage, (**F**) tumor grade, (**G**) nodal metastasis status, and (**H**) TP53 mutation status.

Furthermore, we analyzed the association between PPM1G expression and clinicopathological characteristics in the UALCAN database (using TCGA-LIHC data). The results showed elevated expression levels of PPM1G in LIHC patients compared with normal controls in subgroup analysis based on disease stage, grade, sex, age, race, histological subtype, and TP53 mutational status (Figure 3).

### Correlation of PPM1G promoter methylation with clinical characteristics in LIHC

**Figure 4:**
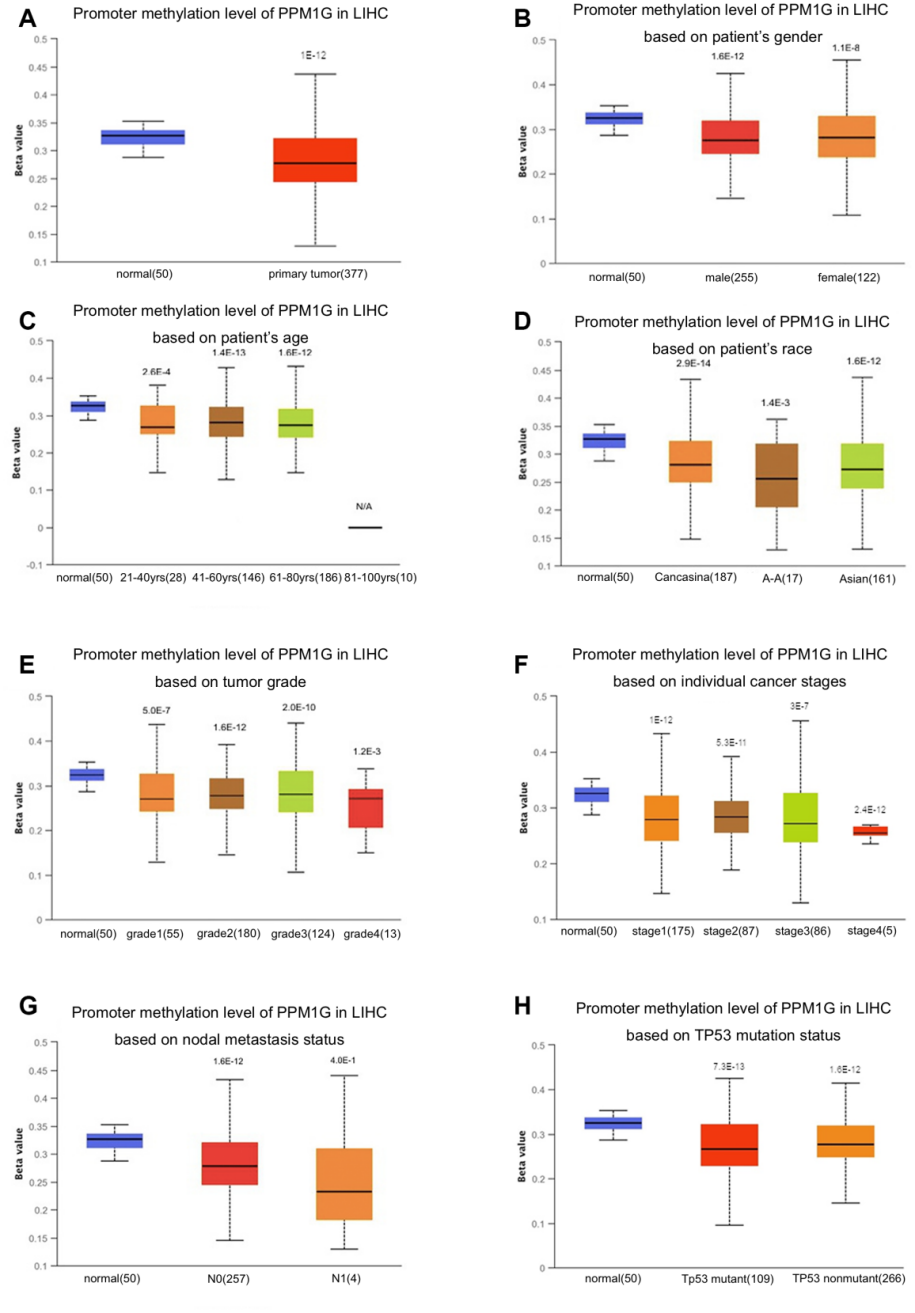
**Relationship of PPM1G promoter methylation level with clinical characteristics in LIHC (UALCAN).** Box-whisker plots showing the relationship between PPM1G promoter methylation in LIHC (different color plots) and normal (blue plots) tissues and patient characteristics: (**A**) normal vs. primary tumor, (**B**) patient sex, (**C**) patient age, (**D**) patient race, (**E**) tumor grade, (**F**) individual cancer stage, (**G**) nodal metastasis status, and (**H**) TP53 mutation status. The beta value indicates the level of DNA methylation (ranging from 0 (unmethylated) to 1 (fully methylated)).

Promoter methylation is one of the most important regulatory factors of gene expression. Subgroup analysis of multiple clinicopathological features of TCGA-LIHC samples via the UALCAN database indicated that the promoter methylation of PPM1G was significantly lower in LIHC patients than in normal controls in subgroups based on stage, grade, sex, age, and ethnicity (Figure 4).

### Mutations and CNV of the PPM1G gene in LIHC

**Figure 5:**
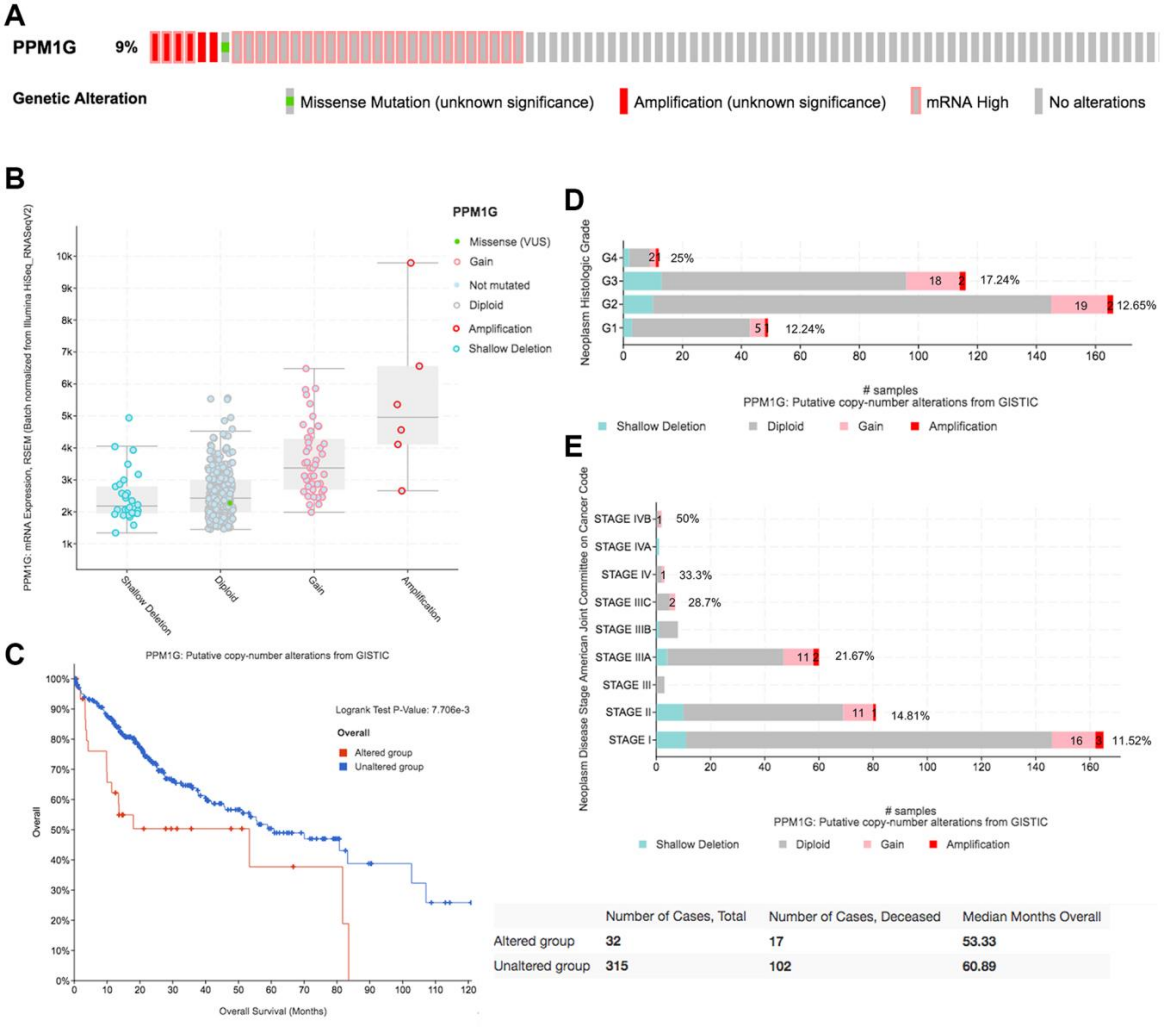
**CNAs of PPM1G in LIHC (cBioPortal).** (**A**) OncoPrint plot of PPM1G alterations in the LIHC cohort. The different types of genetic alterations are highlighted in different colors. (**B**) Correlation between PPM1G expression and CNAs in LIHC. The PPM1G amplification (AMP) group had significantly increased expression. (**C**) Distribution of PPM1G CNV frequency across different grade subgroups. (**D**) Distribution of PPM1G CNV frequency across different stage subgroups. The percentage on the right of the bar indicates the proportion of patients with PPM1G gain or AMP in all subgroups of patients. (**E**) PPM1G CNV affects overall survival.

We then evaluated the type and frequency of PPM1G CNVs in LIHC based on DNA sequencing data from cBioPortal (using PanCancer Atlas data from TCGA) [26]. PPM1G was altered in 32 of 348 (9%) LIHC patients (Figure 5A), including upregulation of mRNA in 29 patients (8.3%), amplification (AMP) in 6 patients (1.7%), and mutation in 1 patient (0.3%). Both PPM1G AMP and mRNA upregulation were found in 4 patients. Therefore, upregulation of mRNA is the most general type of alteration affecting PPM1G in LIHC.

PPM1G AMP led to high PPM1G expression levels (Figure 5B). Compared with that in the diploid group, the expression levels of PPM1G in the gain and AMP groups were upregulated. In addition, we determined the PPM1G CNV frequency distribution in patients stratified based on stage and grade (Figure 5C, 5D). The results demonstrated that PPM1G expression was positively associated with the CNV level in LIHC. Furthermore, PPM1G CNV was significantly correlated with overall survival (OS) in LIHC (Figure 5E) but not significantly associated with disease-free survival (DFS).

### PPM1G expression is associated with survival

**Figure 6:**
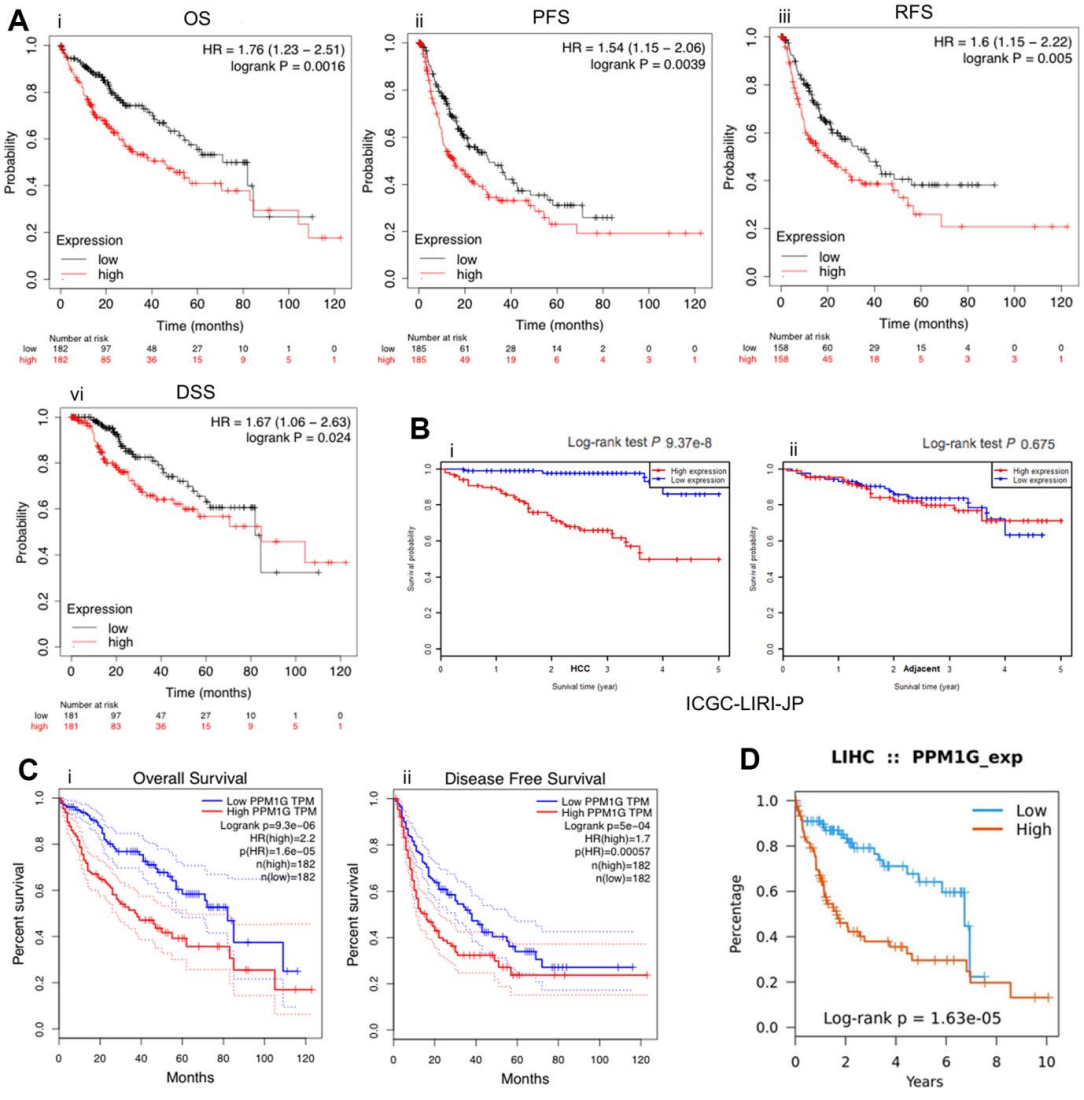
**PPM1G is associated with survival.** Survival curves representing the survival of patients with LIHC with high (red) and low (black) PPM1G expression. (**A**) Overall survival (OS), progression-free survival (PFS), recurrence-free survival (RFS), and disease-specific survival (DSS) (kmplot). (**B**) OS in the ICGC-LIRI-JP cohort (**i**) and adjacent cohort (**ii**) (HCCDB database). (**C**) OS and disease-free survival (DFS) in LIHC patients (GEPIA). (**D**) OS in LIHC patients (TISIDB).

To reveal the prognostic value of PPM1G expression in LIHC, KM survival curves were used to examine the relationship between PPM1G expression and the survival status of patients with LIHC. LIHC patients were separated into two groups according to the median value of the PPM1G expression level in each cohort. High expression was significantly positively associated with poor OS, progression-free survival (PFS), recurrence-free survival (RFS), and disease-specific survival (DSS) according to the Kaplan–Meier (KM) plotter web tool (which uses TCGA, GEO, and European Genome-phenome Archive (EGA) data) (Figure 6A). Similarly, in GEPIA, the low expression group had significantly better OS and DFS than the high expression group (HR>1, p<0.05) (Figure 6C). We found a similar tendency in the Tumor-Immune System Interactions Database (TISIDB) (which uses TCGA data) (Figure 6D). As a verification, in another independent cohort (ICGC-LIRI-JP), the high expression group had significantly poorer OS than the low expression group (p<0.001) in LIHC (Figure 6D).

### Coexpressed genes of PPM1G in LIHC

**Figure 7:**
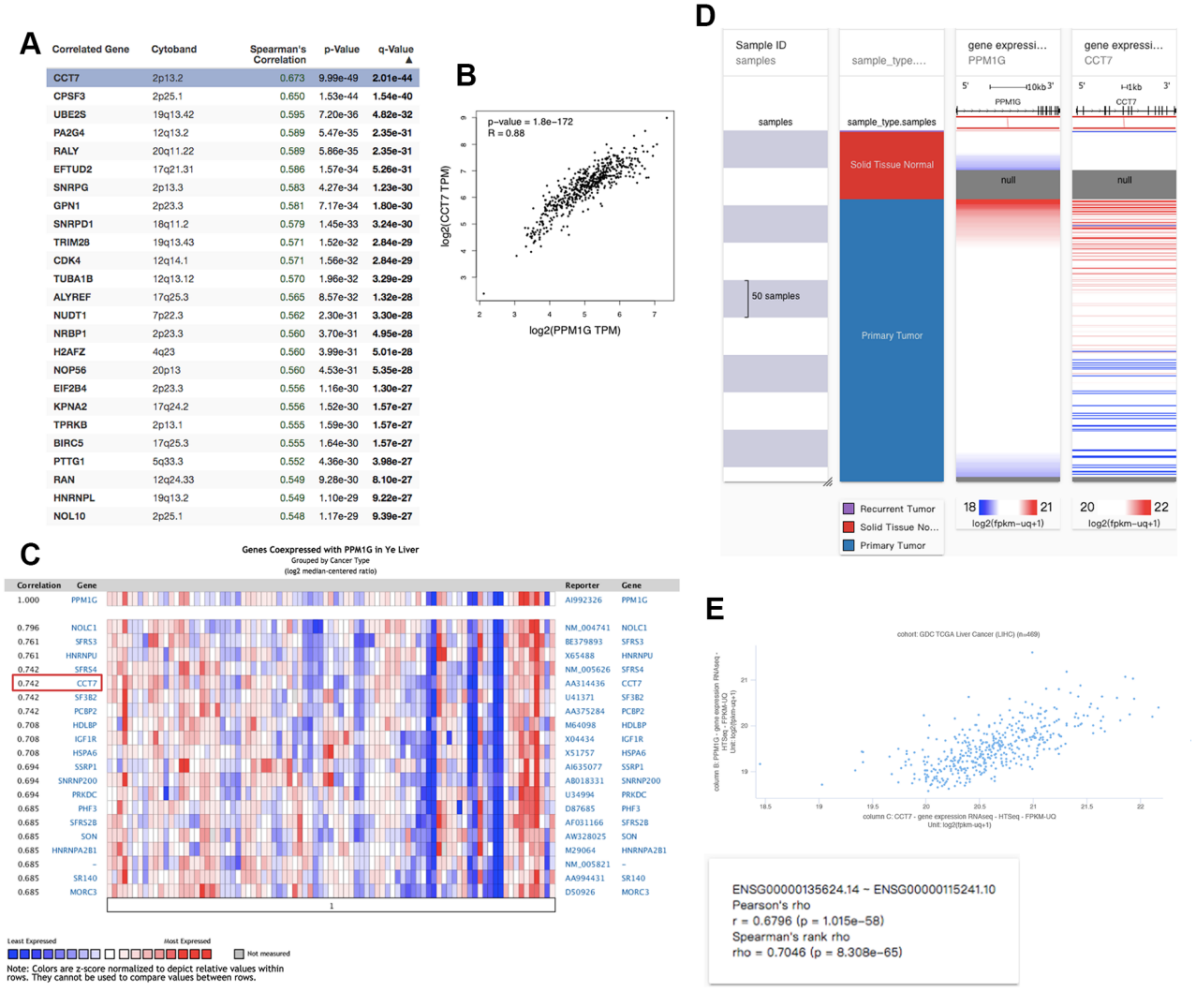
Coexpression profile of PPM1G in LIHC: (**A**) PPM1G coexpressed genes in LIHC identified by Spearman’s test (cBioPortal). (**B**) Coexpression analysis of PPM1G and CCT7 in LIHC (GEPIA). (**C**) PPM1G coexpressed genes (Oncomine). (**D**) Heat map of PPM1G and CCT7 mRNA expression across LIHC samples (UCSC Xena). (**E**) Coexpression analysis of PPM1G and CCT7 transcription in LIHC (UCSC Xena).

We identified PPM1G coexpressed genes in the LIHC cohort via the cBioPortal database to explore the biological significance of PPM1G. We identified the top 26 (ranked according to Spearman’s correlation coefficient) coexpressed genes of PPM1G in the LIHC cohort, including CCT7, CPSF3, UBE2S, EFTUD2, RALY, PA2G4, SNRPG, GPN1, SNRPD1, TUBA1B, TRIM28, CDK4, ALYREF, NRBP1, NUDT1, H2AFZ, NOP56, EIF2B4, KPNA2, BIPTTG1, TPRK, HNRNPL, RAN, and SNRPB (Figure 7A). CCT7 expression was most correlated with PPM1G (r=0.674). The correlation between PPM1G and CCT7 was verified in an independent cohort (Ye Liver, GSM5328, n=87) via the Oncomine (log2 median-centered ratio=0.742, P<1E-4, fold change>2) (Figure 7C), GEPIA (r=0.88) (Figure 7B) and the University of California Santa Cruz (UCSC) Xena (Pearson r=0.68; Spearman r=0.70) platforms (Figure 7D). Furthermore, we found that CCT7 was overexpressed in LIHC (Supplementary Figure 1) and associated with OS and RFS in LIHC patients via GEPIA (Supplementary Figure 2). Notably, PPM1G and CCT7 may be involved in signal transduction pathways in LIHC.

### Functional and pathway enrichment analyses of PPM1G

**Figure 8:**
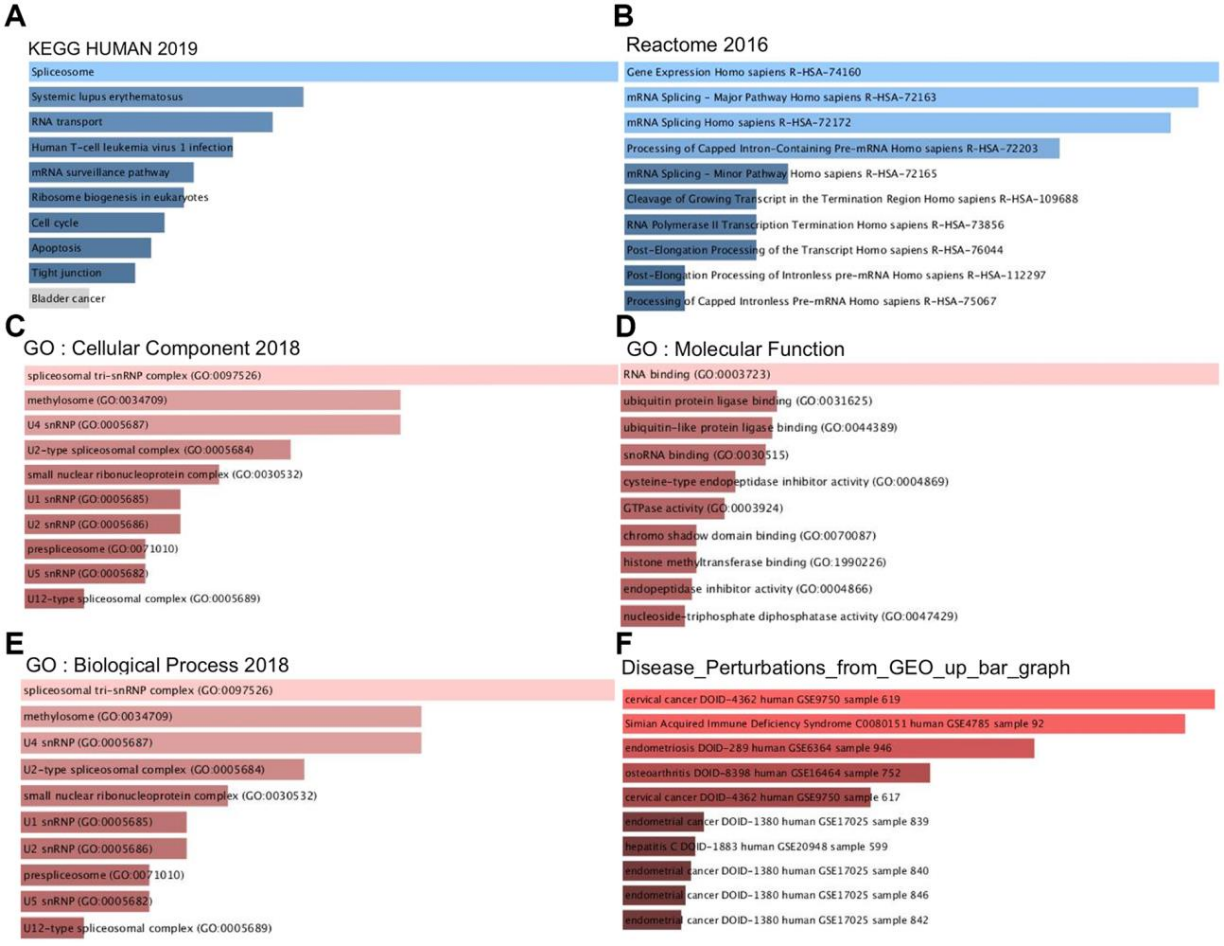
**Signaling pathways of PPM1G coexpressed genes (Enrichr).** These figures show the gene ontology (GO) and signaling pathways of PPM1G in LIHC. (**A**) KEGG pathways (2019). (**B**) REACTOME pathways (2016). (**C**) GO cellular component (CC) (2018) terms. (**D**) GO molecular function (MF) (2018) terms. (**E**) GO biological process (BP) (2018) terms. (**F**) Bar graph showing the “Disease perturbations from GEO up ” gene set. The bar graph represents the ratio of the percent composition of terms in proteomic data to percent composition in the genome annotation. The length of the bar represents the significance of that specific gene set or term. The brighter the color is, the more significant the term.

The PPM1G coexpressed genes were subjected to functional and pathway enrichment analyses with the Enrichr online tool (Figure 8). The top 10 Kyoto Encyclopedia of Genes and Genomes (KEGG) pathways were pathways related to spliceosomes, systemic lupus erythematosus, RNA transport, human T cell leukemia virus 1 infection, the mRNA surveillance pathway, ribosome biogenesis in eukaryotes, the cell cycle, apoptosis, and tight junctions. The top 10 REACTOME pathways were pathways related to gene expression, mRNA splicing, processing of capped introns, cleavage of the growing transcript in the termination region, RNA polymerase II transcription termination, post-elongation processing of the transcript, post-elongation processing of intronless pre-mRNA, and processing of capped intronless pre-mRNA. Next, GO analysis was used to determine the relevant biological processes, molecular functions, and cellular components. PPM1G and its positively correlated genes were mainly related to the spliceosomal tri-snRNP complex and RNA binding.

To discover potential diseases related to PPM1G and its coexpressed genes, we studied the “Disease Perturbations from GEO up” gene set of Enrichr. PPM1G and its coexpressed genes were enriched in cervical cancer, simian acquired immune deficiency syndrome, endometriosis, osteoarthritis, endometrial cancer, and hepatitis.

### Kinases involved in PPM1G networks in LIHC

To determine the kinases correlated with PPM1G, we studied the “Kinase Gene Enrichment” gene set in Enrichr. The top 5 kinases were PLK1, AURKB, CDK4, CDK2, and PKMYT1 (Table 1). All these kinases were highly expressed in LIHC, except for CDK2 (Supplementary Figure 3). In addition, the gene expression of all these kinases was significantly correlated with clinical outcome of LIHC (Supplementary Figure 4).

**Table 1:** The kinases networks of PPM1G in LIHC.

**Enriched category**	**Genes**	**FDR**	**Combined score**
**Kinases**	PLK1	8.407E-17	1556.87
	AURKB	1.093E-11	870.39
	CDK4	7.290E-12	870.39
	CDK2	2.569E-10	692.23
	PKMYT1	2.055E-10	692.23

### Gene mutations cooccurring with PPM1G alterations in LIHC

**Figure 9:**
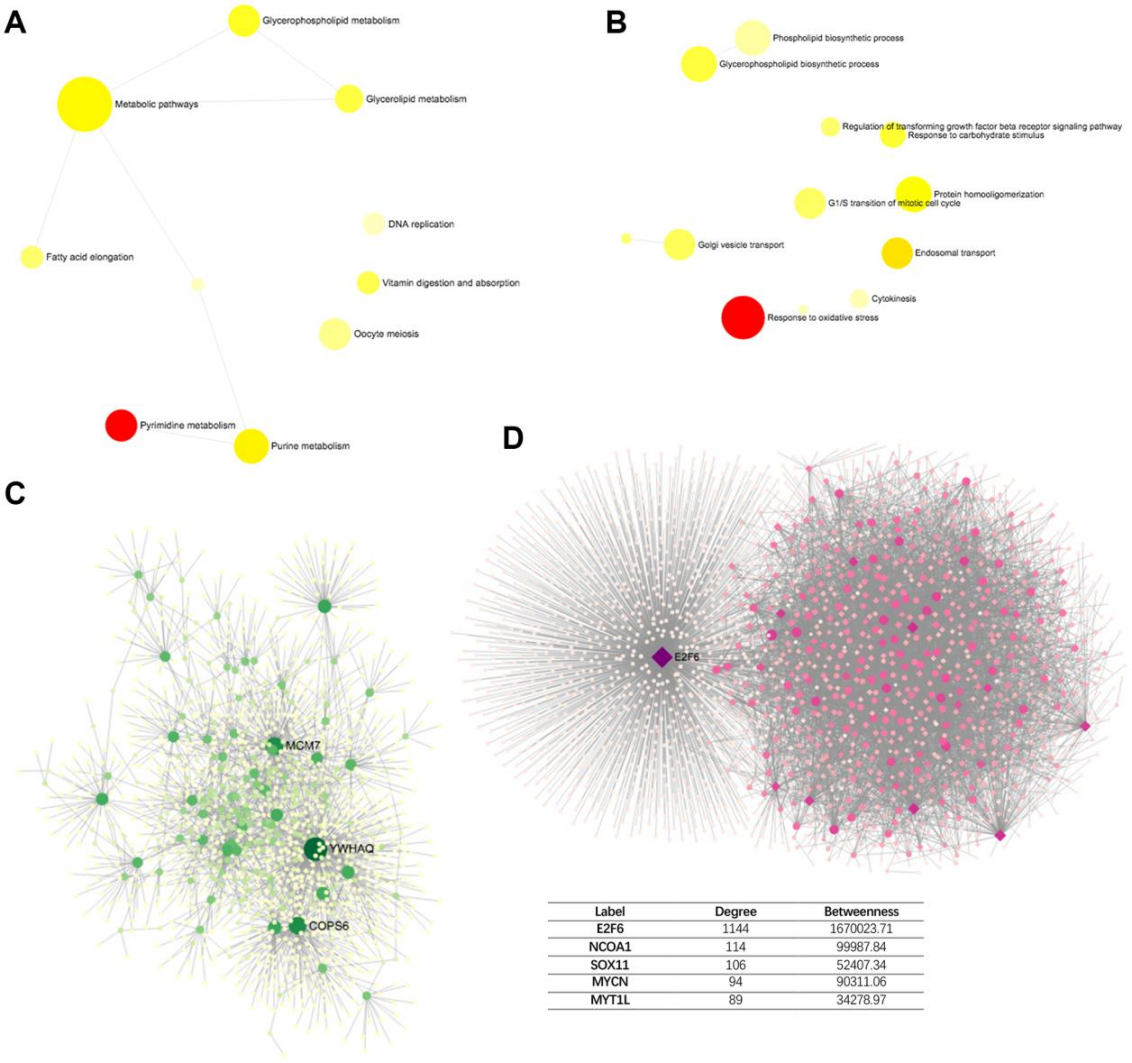
**Network of PPM1G AMP co-altered genes in LIHC (DifferentialNet).** (**A**) Significant KEGG pathways. (**B**) Significant GO BP terms. (**C**) Significant liver-specific PPI network. (**D**) Significant TF-miRNA coregulatory network.

To explore the common functional genetic alterations associated with PPM1G, we determined the profile of mutations cooccurring with PPM1G alterations in LIHC via cBioPortal. A total of 260 gene alterations significantly cooccurred with PPM1G AMP (Supplementary Table 1). The top 3 most frequent alterations were alterations of ABHD1 (29.41%), ATRAID (29.41%) and CAD (29.41%). The genes with cooccurring alterations were significantly enriched in pyrimidine metabolism and purine metabolism and metabolic pathways according to the KEGG analysis (Figure 9A). The GO analysis showed that PPM1G co-altered genes participated primarily in the response to oxidative stress, endosomal transport, and protein homooligomerization (Figure 9B).

Next, to identify the hub genes of PPM1G, we investigated the protein-protein interaction (PPI) network of the proteins encoded by the PPM1G co-altered genes as generated via the DifferentialNet database (Figure 9C) [27]. The top 3 hub genes were YWHAQ, COPS6 and MCM7. Overexpression of YWHAQ in primary tumors was associated with poor prognosis in LIHC patients [28]. MCM7 promotes liver cancer progression through cyclin D1-dependent signaling [29]. The REACTOME analysis of PPM1G AMP revealed cell cycle, chromosome maintenance, immune system, adaptive immune system, etc., as related terms (Table 2).

**Table 2:** Reactome annotation of PPM1G co-occurrence PPI network.

**Pathway**	**Total**	**Expected**	**Hits**	**P.Value**	**FDR**
**Cell Cycle**	508	59	144	4.17E-27	5.85E-24
**Cell Cycle, Mitotic**	411	47.8	111	5.36E-19	3.47E-16
**Chromosome Maintenance**	124	14.4	53	7.42E-19	3.47E-16
**Deposition of New CENPA-containing Nucleosomes at the Centromere**	65	7.55	34	8.34E-16	2.34E-13
**Nucleosome assembly**	65	7.55	34	8.34E-16	2.34E-13
**Immune System**	1140	132	204	2.91E-12	6.79E-10
**Adaptive Immune System**	654	76	131	3.60E-11	7.22E-09
**Nuclear Receptor transcription pathway**	53	6.16	25	1.10E-10	1.93E-08
**Mitotic G2-G2/M phases**	105	12.2	37	1.54E-10	2.40E-08
**Telomere Maintenance**	72	8.37	29	4.00E-10	5.61E-08

Furthermore, a transcription factor-miRNA coregulatory network of the PPM1G co-altered genes was assembled based on the RegNetwork repository (Figure 9D) [30]. The top 3 transcription factors (TFs) were E2F6, NCOA1 and SOX11. A previous study indicated that E2F6, playing a crucial role in the control of the cell cycle, was enriched in basophils, and alterations in its DNA sequence were associated with poor prognosis [31]. The liver-specific PPI network functional annotation implied that PPM1G AMP is involved in the immune response.

### Regulation of immune molecules by PPM1G in LIHC

**Figure 10:**
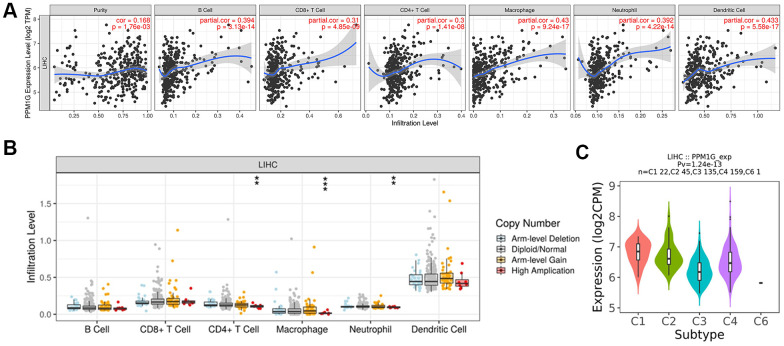
**Correlations of PPM1G expression with the immune infiltration level and immune subtypes in LIHC.** (**A**) PPM1G expression was significantly correlated with tumor purity and the levels of infiltrating B cells, CD8+ T cells, CD4+ T cells, macrophages, neutrophils, and dendritic cells in LIHC (TIMER). (**B**) Influence of PPM1G CNV on the levels of infiltrating immune cells in LIHC (TIMER). (**C**) Distribution of PPM1G expression across immune subtypes in LIHC (TISIDB). The different color plots represent the six immune subtypes (C1: wound healing; C2: IFN-gamma dominant; C3: inflammatory; C4: lymphocyte-depleted; C5: immunologically quiet; and C6: TGF-b dominant).

To further explore the immune-related functions of PPM1G, we analyzed the association between PPM1G and the tumor microenvironment in TIMER. PPM1G expression was significantly correlated with tumor purity (Spearman’s r=0.168, p=1.76E-03) and dominant immune cells (Figure 10A). PPM1G CNV was significantly associated with the infiltration levels of CD4+ T cells, macrophages and neutrophils (Figure 10B).

Then, we used TISIDB to evaluate the association between immune subtypes and PPM1G expression (Figure 10C). Five immune subtypes were identified in LIHC (C1-C4 and C6 subtypes). The lymphocyte-depleted (C4) subtype accounted for the largest proportion of all subtypes (n=159, 44%), followed by the inflammatory (C3) subtype (n=135, 37%). PPM1G expression was lower in C3 than in the C4 subtype. Thus, the results above indicated that most LIHC patients with PPM1G expression were in C3 or C4 immune subtype.

## DISCUSSION

A previous study indicated that PPM phosphatases were linked to regulating various cell functions, such as cell cycle control, cell differentiation, immune response and cell metabolism. Mutations and overexpression of PPM phosphatase genes have been observed in various cancers. As a member of the PPM family, PPM1G, a Mg2+/Mn2+-dependent nuclear serine/threonine phosphatase, plays an important role in regulating pre-mRNA splicing, DNA damage responses [13] and carcinogenesis [32]. Splicing is a crucial step in eukaryotic gene expression. Many molecular alterations observed in cancers come from modifications in the splicing process [12]. Splicing is expected to be a major source of untapped molecular targets for precision oncology [33]. PPM1G plays a fundamental role in increasing liver fibrosis by negatively regulating the effect of WWP2 on Notch3 degradation [4]. More than 80% of hepatocellular carcinoma tumors develop in fibrotic or cirrhotic livers, suggesting an important role of liver fibrosis in the premalignant environment of the liver [3]. Thus, PPM1G might relate to the pathogenesis of LIHC.

To reveal the expression profile of PPM1G in LIHC, we analyzed over 3,400 clinical samples from LIHC studies in 7 countries and regions. The results confirmed that PPM1G mRNA levels, protein levels and CNVs were significantly higher in LIHC than in normal adjacent control samples (Figure 2A). The enrichment analysis showed that PPM1G coexpressed genes were enriched in pathways related to spliceosomes, the cell cycle, apoptosis and other processes. Previous research has suggested that many mutations are linked to alterations in splicing in diverse types of cancers [12]. Apoptosis, one of the most studied forms of programmed cell death, is a fundamental feature in several biological processes, such as embryonic development, immune responses, and cell turnover [12]. In cancer, apoptosis pathways are usually dysregulated [34]. The splicing, cell cycle and apoptosis processes are consistent with tumorigenesis. Furthermore, upregulation of PPM1G expression was significantly associated with poor survival. All the above results suggest that PPM1G affects the prognosis of LIHC by dysregulating splicing, the cell cycle and apoptosis processes.

Kinases regulate the gene expression process as regulatory factors involved in hepatocellular carcinogenesis. Therefore, we investigated the kinase and transcription factor network of PPM1G. PLK1, AURKB, CDK4, CDK2, PKMYT1, and PBK were enriched in the network based PPM1G and its coexpressed genes in LIHC. These kinases regulate cell proliferation and differentiation. PLK1 plays an important role in the initiation, maintenance and completion of mitosis, and it is the main driving force of cancer cell growth and proliferation [35]. The PLK1 inhibitor volasertib has entered phase III clinical trials and has shown considerable prospects in clinical studies [36]. The relationship between PLK1 and liver cancer has recently been confirmed [37]. In addition, uncontrolled cellular proliferation mediated by dysregulation of the cell cycle machinery and activation of cyclin-dependent kinases to promote cell cycle progression is a pathological process that lies at the heart of cancer development [38]. It has been confirmed that the inhibition of CDK4 and CDK2 triggers antitumor activity in some tumors. Some inhibitors of CDK4/6 have been approved for use in combination with nonsteroidal aromatase inhibitors for the first-line treatment of breast cancer in postmenopausal women, and PFS can be increased by 40-45% [39, 40]. PBK overexpression promotes the metastasis of hepatocellular carcinoma by activating the ETV4-uPAR signaling pathway [41]. In LIHC, PPM1G may upregulate the above kinases to affect chromatin remodeling, mRNA splicing, DNA damage and cell cycle progression, leading to a poor outcome.

Next, to investigate DNA variations of PPM1G, we constructed a gene network of genes co-altered with PPM1G AMP in LIHC. The enriched functional terms related to the gene network included the cell cycle, chromosome maintenance, the immune system, and the adaptive immune system.

In recent years, increasing attention has been given to the tumor immune microenvironment, which has changed precision medicine therapy for cancer. The network of PPM1G AMP co-alterations was enriched in several immune functions. Therefore, we hypothesized that the expression of PPM1G might be linked to the tumor immune microenvironment in LIHC. We examined the immune microenvironment in which PPM1G is located. The results showed that PPM1G was significantly associated with tumor purity and the infiltration of dominant immune cells. The mechanism of PPM1G in the tumor immune microenvironment deserves further exploration. The outcome of immunotherapy for cancer mainly depends on the immune response mediated by T cells (which is affected by alternative splicing). Thus, the correlation between PPM1G expression and the function of T cells needs further confirmation.

Immune subtypes were found to have potential therapeutic and prognostic implications for cancer management [42]. Therefore, we explored the relationship between PPM1G expression and liver cancer immune subtypes. The results showed that patients with PPM1G expression mostly C3 or C4 immune subtype. The C4 subtype was associated with poor outcomes. The C3 subtype was related to the most favorable prognosis. Interestingly, the expression level of PPM1G in C4 immune subtype was higher than that in C3 immune subtype. Higher expression of PPM1G may be related to shorter survival, which is consistent with the PPM1G survival curves. However, the subtype research was conducted across various cancers, and the results may differ in LIHC. Thus, hepatocellular carcinoma immune subtypes need further exploration. The above results suggest that PPM1G may be correlated with the tumor immune microenvironment.

## CONCLUSIONS

In this study, we systematically analyzed the expression profile, mutation profile, survival, regulation networks, epigenetics, functional pathways and immune infiltration related to PPM1G in LIHC. Furthermore, we found that PPM1G expression upregulation was positively correlated with poor prognosis in LIHC. The transcription of PPM1G can be regulated by promoter methylation, kinases and CNVs. Overexpression of PPM1G may be involved in mRNA splicing and cell cycle processes. In addition, PPM1G might play a role in the immune microenvironment of LIHC. These results suggest that PPM1G may be a prognostic biomarker for survivals of LIHC patients, and PPM1G may play a potential novel immune regulatory role in the tumor immunity in LIHC.

## MATERIALS AND METHODS

### Evaluation of PPM1G expression in various cancers

PPM1G expression profiles in various cancers were analyzed with the GEPIA database (http://gepia.cancer-pku.cn/) [43], UALCAN (http://ualcan.path.uab.edu) [44] and the GENT database (http://gent2.appex.kr/gent2/) [45]. GEPIA and UALCAN are based on TCGA data. In GEPIA, PPM1G expression in tumor samples in TCGA was compared to that in adjacent normal samples via Genotype-Tissue Expression (GTEx) [46]. GENT data comes from the GPL 570 clinical data from 29 cancer types of GEO database. Analyses were carried out with the default settings in all the above databases.

### Evaluation of PPM1G expression in LIHC

The PPM1G transcription patterns in LIHC and normal liver tissues were compared via HCCDB (http://lifeome.net/database/hccdb/home.html) [47], Oncomine 4.5 (https://www.oncomine.org/) [48] and HPA (https://www.proteinatlas.org/) [49]. The HCCDB database includes data from GEO [50–52], TCGA and ICGC [53]. For the Oncomine analysis, the following thresholds were used: p-value of 1E-4, fold change of 1.5, and gene ranking of all. In the HPA database, PPM1G protein expression in LIHC and normal liver tissues was assessed in immunohistochemistry images.

### Analysis of the correlations of PPM1G expression and promoter methylation with various clinical characteristics

We investigated the correlations of PPM1G expression and promoter methylation with clinicopathological characteristics, including cancer stage, tumor grade, sex, age, race, histological subtype, and TP53 mutational status, via UALCAN with the default settings [44].

### Analysis of PPM1G mutations and CNVs in LIHC

PPM1G mutations and CNVs were analyzed with TCGA samples by using cBioPortal datasets (http://cbioportal.org) [54]. Somatic copy number alterations (CNAs) were identified in RNA-seq data by the Genomic Identification of Significant Targets in Cancer (GISTIC) algorithm with the default settings, and mRNA expression data were plotted using the cBioPortal website. The OncoPrint plot displayed an overview of PPM1G genetic alterations per sample.

### Analysis of associations between PPM1G expression and patient survival in LIHC

The associations between patient survival and PPM1G expression in LIHC were visualized via KM plotter (http://kmplot.com/analysis/) [55] (which uses TCGA, GEO, and EGA data), GEPIA(which uses TCGA data) [43] and TISIDB (http://cis.hku.hk/TISIDB/)(which uses TCGA data) [56], and the results were verified in HCCDB(which uses ICGC-LIRI-JP data) [47]. The KM plotter platform was used to quickly evaluate disease prognosis parameters, including OS, PFS, RFS and DSS, according to the median cutoff values.

### Profiling of PPM1G coexpressed genes

The top 25 PPM1G coexpressed genes were identified via cBioPortal [54]. Then, we used GEPIA, UCSC Xena (http://xena.ucsc.edu/) [57] and Oncomine (an idependent database, which is different from cBioPortal, GEPIA and UCSC) to verify the relationship between PPM1G and CCT7. UCSC Xena is a cancer genomics data analysis platform that supports the visualization and analysis of various omics data from cancer samples.

### Prediction of the signaling pathways and functions of PPM1G and its coexpressed genes

We performed GSEA with KEGG, REACTOME, GO, relevant disease and kinase gene sets via Enrichr (https://amp.pharm.mssm.edu/Enrichr) [58] to identify pathways and functions related to PPM1G and its coexpressed genes. PPM1G coexpression was analyzed statistically using Pearson’s correlation coefficient.

### Visualization of the PPM1G CNV co-altered gene network

We identified PPM1G CNV co-altered genes via cBioPortal [54]. Networks were generated with the NetworkAnalyst 3.0 tool (https://www.networkanalyst.ca/) [59], including PPI networks and gene regulatory networks. The functional enrichment analysis was similar to that used by ClueGO and Enrichment Map.

### Investigation of immune infiltrates and immune subtypes related to PPM1G in LIHC

The correlations of PPM1G and its coexpressed genes with immune infiltrates were explored via TIMER (https://cistrome.shinyapps.io/timer/), a comprehensive resource for the systematic analysis of immune infiltrates across diverse cancer types from TCGA data (10,897 samples across 32 cancer types) [60]. We analyzed the correlations of PPM1G expression with infiltrating immune cells, including B cells, CD4+ T cells, CD8+ T cells, neutrophils, macrophages, and dendritic cells, and tumor purity. Then, we analyzed the relationship between immune subtype and PPM1G via TISIDB. TISIDB is a web portal for assessing tumor and immune system interactions that integrates multiple heterogeneous data types. A threshold of p < 0.05 indicated significance of the correlation.

### Data availability statement

The datasets used and/or analyzed in current study can be obtained from the corresponding author on reasonable request.

## Supplementary Material

Supplementary Figures

Supplementary Table 1
