# Supplementary material for: Prognostic and immunological potential of PPM1G in hepatocellular carcinoma
Source: Aging (Albany NY). 2021 May 5;13(9):12929–54. doi: 10.18632/aging.202964 (PMC8148464; doi:10.18632/aging.202964)
Supplement: Supplementary Figures [file aging-13-202964-s001.pdf]

SUPPLEMENTARY FIGURES

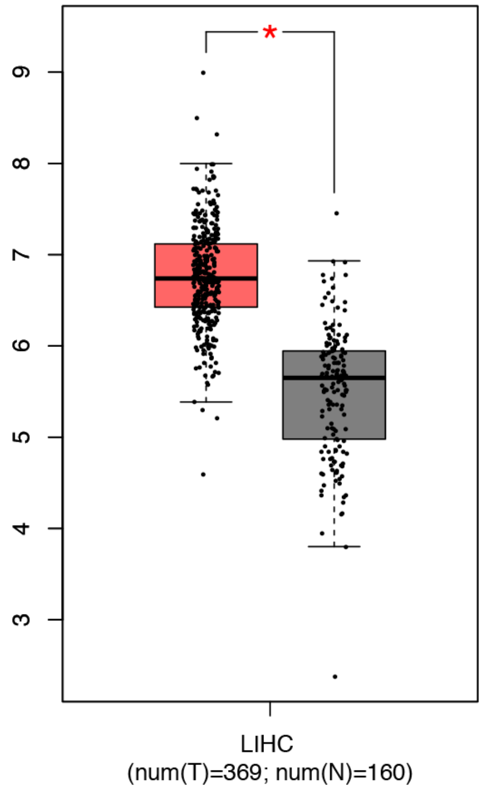

Supplementary Figure 1. CCT7 expression in LIHC (GEPIA). The box plots represent CCT7 mRNA expression in LIHC (red plot) and normal (blue plot) tissues.

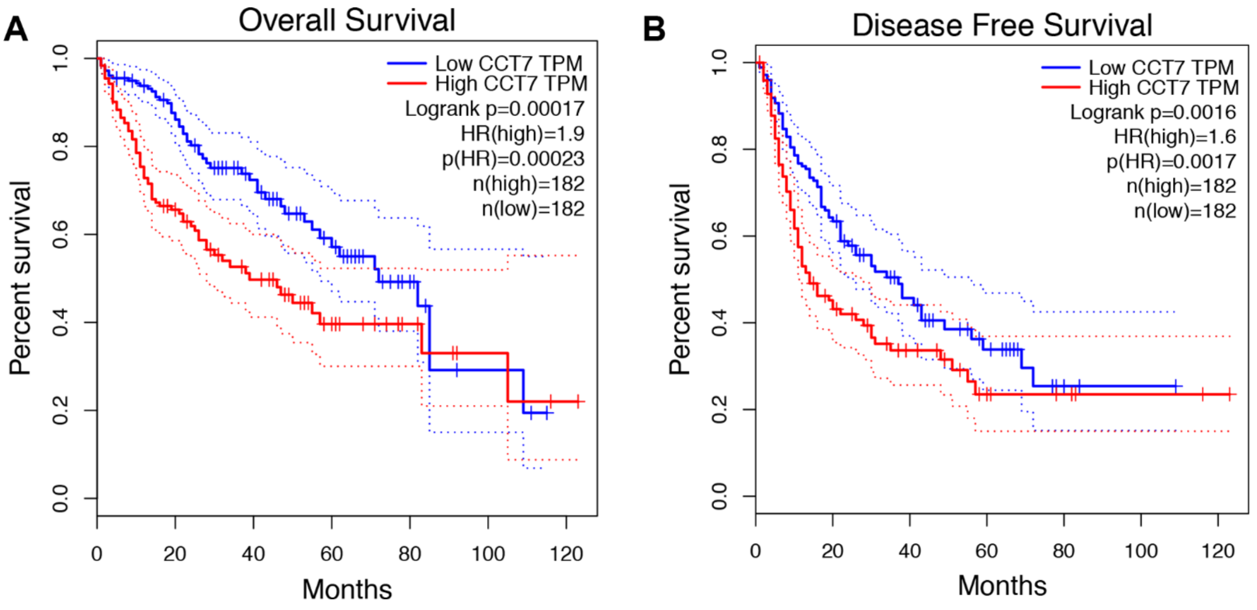

Supplementary Figure 2. Association between survival and CCT7 expression level in LIHC (GEPIA). The survival curves demonstrate the survival of patients with high (red) and low (blue) CCT7 expression in LIHC. (**A**) Overall survival (OS). (**B**) disease-free survival (DFS).

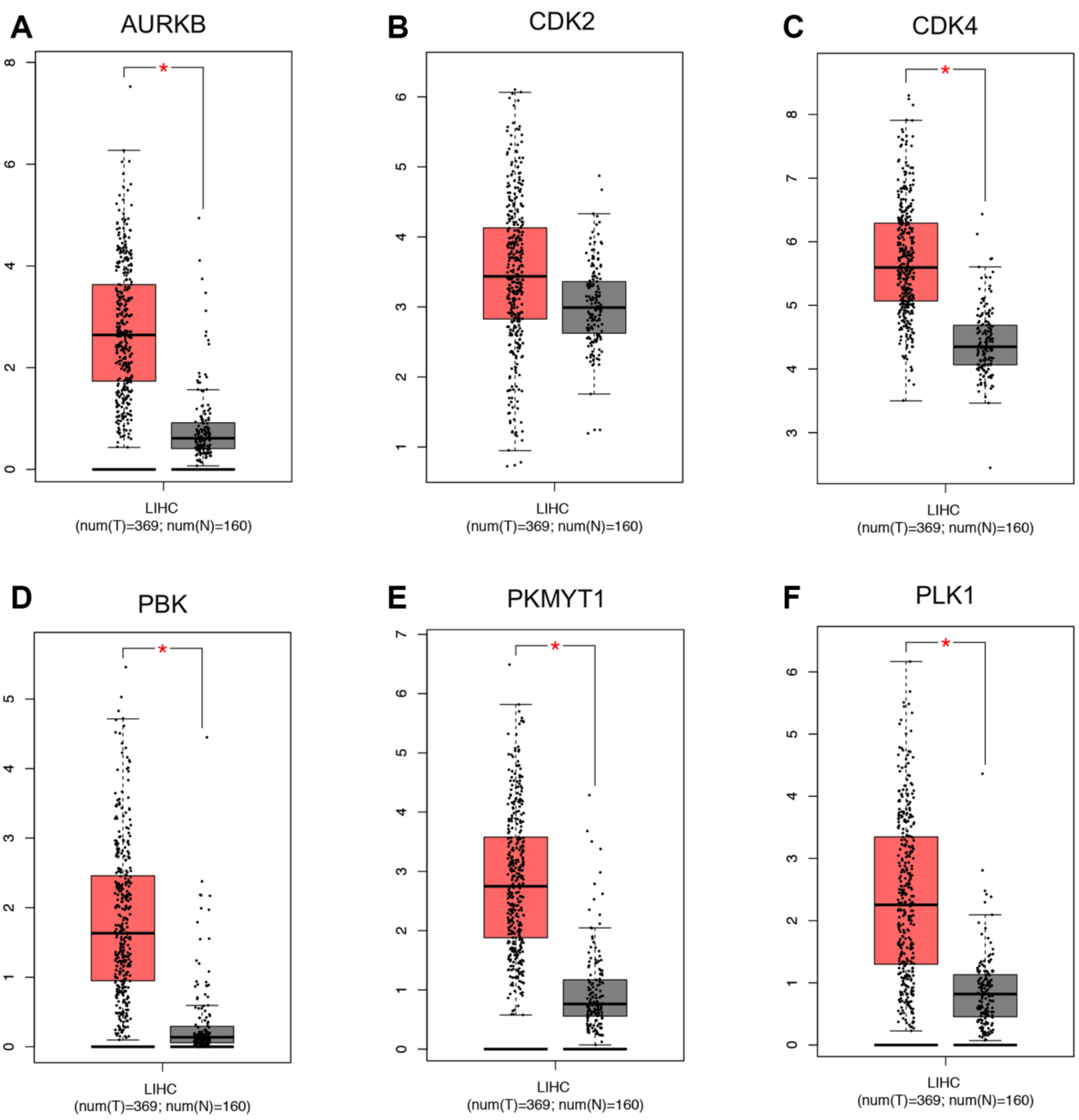

Supplementary Figure 3. Expression level of kinase genes related to PPM1G in LIHC (GEPIA). The box plots show the mRNA expression of kinase genes: (**A**) AURKB, (**B**) CDK2, (**C**) CDK4, (**D**) PBK, (**E**) PKMYT1 and (**F**) PLK1 in LIHC (red plot) and normal (blue plot) tissues.

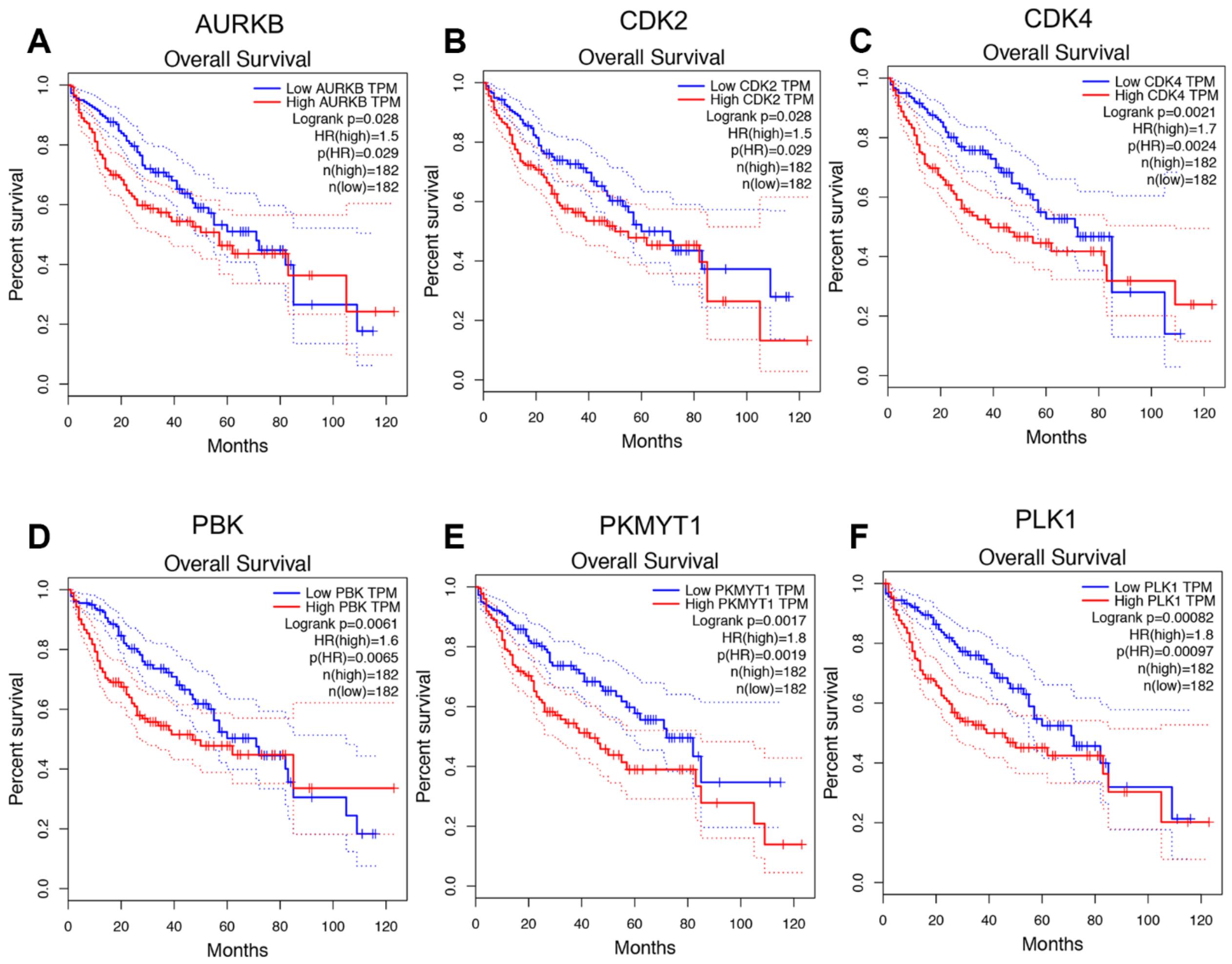

Supplementary Figure 4. Association between OS and the expression of kinase genes related to PPM1G in LIHC (GEPIA). The survival curves demonstrate the survival of patients with high (red) and low (blue) expression of PPM1G-related kinase genes: (**A**) AURKB, (**B**) CDK2, (**C**) CDK4, (**D**) PBK, (**E**) PKMYT1 and (**F**) PLK1 in LIHC.
