# Supplementary material for: Prognostic and immunological potential of PPM1G in hepatocellular carcinoma
Source: Aging (Albany NY). 2021 May 5;13(9):12929–54. doi: 10.18632/aging.202964 (PMC8148464; doi:10.18632/aging.202964)
Supplement: Supplementary Table 1 [file aging-13-202964-s002.pdf]

SUPPLEMENTARY TABLE

Supplementary Table 1. Top 40 co-occurrence genes of PPM1G alterations in LIHC (cBioportal).

No	Gene	Cytoband	Alteration	Altered group	Log ratio	p-Value	q-Value	Enriched in
1	ABHD1	2p23.3	Amp	10 (29.41%)	>10	1.48E-11	4.65E-08	Altered group
2	ATRAID	2p23.3	Amp	10 (29.41%)	>10	1.48E-11	4.65E-08	Altered group
3	CAD	2p23.3	Amp	10 (29.41%)	>10	1.48E-11	4.65E-08	Altered group
4	CGREF1	2p23.3	Amp	10 (29.41%)	>10	1.48E-11	4.65E-08	Altered group
5	PREB	2p23.3	Amp	10 (29.41%)	>10	1.48E-11	4.65E-08	Altered group
6	PRR30	2p23.3	Amp	10 (29.41%)	>10	1.48E-11	4.65E-08	Altered group
7	SLC30A3	2p23.3	Amp	10 (29.41%)	>10	1.48E-11	4.65E-08	Altered group
8	SLC5A6	2p23.3	Amp	10 (29.41%)	>10	1.48E-11	4.65E-08	Altered group
9	TCF23	2p23.3	Amp	10 (29.41%)	>10	1.48E-11	4.65E-08	Altered group
10	AGBL5	2p23.3	Amp	9 (26.47%)	>10	2.07E-10	2.93E-07	Altered group
11	DNAJC5G	2p23.3	Amp	9 (26.47%)	>10	2.07E-10	2.93E-07	Altered group
12	DPYSL5	2p23.3	Amp	9 (26.47%)	>10	2.07E-10	2.93E-07	Altered group
13	EMILIN1	2p23.3	Amp	9 (26.47%)	>10	2.07E-10	2.93E-07	Altered group
14	KHK	2p23.3	Amp	9 (26.47%)	>10	2.07E-10	2.93E-07	Altered group
15	MAPRE3	2p23.3	Amp	9 (26.47%)	>10	2.07E-10	2.93E-07	Altered group
16	MPV17	2p23.3	Amp	9 (26.47%)	>10	2.07E-10	2.93E-07	Altered group
17	OST4	2p23.3	Amp	9 (26.47%)	>10	2.07E-10	2.93E-07	Altered group
18	TMEM214	2p23.3	Amp	9 (26.47%)	>10	2.07E-10	2.93E-07	Altered group
19	TRIM54	2p23.3	Amp	9 (26.47%)	>10	2.07E-10	2.93E-07	Altered group
20	UCN	2p23.3	Amp	9 (26.47%)	>10	2.07E-10	2.93E-07	Altered group
21	GDF7	2p24.1	Amp	9 (26.47%)	6.43	1.94E-09	1.66E-06	Altered group
22	LDAH	2p24.1	Amp	9 (26.47%)	6.43	1.94E-09	1.66E-06	Altered group
23	ADCY3	2p23.3	Amp	8 (23.53%)	>10	2.81E-09	1.66E-06	Altered group
24	ATAD2B	2p24.1-p23.3	Amp	8 (23.53%)	>10	2.81E-09	1.66E-06	Altered group
25	CENPA	2p23.3	Amp	8 (23.53%)	>10	2.81E-09	1.66E-06	Altered group
26	CENPO	2p23.3	Amp	8 (23.53%)	>10	2.81E-09	1.66E-06	Altered group
27	EIF2B4	2p23.3	Amp	8 (23.53%)	>10	2.81E-09	1.66E-06	Altered group
28	FAM228A	2p23.3	Amp	8 (23.53%)	>10	2.81E-09	1.66E-06	Altered group
29	FAM228B	2p23.3	Amp	8 (23.53%)	>10	2.81E-09	1.66E-06	Altered group
30	FKBP1B	2p23.3	Amp	8 (23.53%)	>10	2.81E-09	1.66E-06	Altered group
31	GTF3C2	2p23.3	Amp	8 (23.53%)	>10	2.81E-09	1.66E-06	Altered group
32	ITSN2	2p23.3	Amp	8 (23.53%)	>10	2.81E-09	1.66E-06	Altered group
33	MFSD2B	2p23.3	Amp	8 (23.53%)	>10	2.81E-09	1.66E-06	Altered group
34	NCOA1	2p23.3	Amp	8 (23.53%)	>10	2.81E-09	1.66E-06	Altered group
35	PFN4	2p23.3	Amp	8 (23.53%)	>10	2.81E-09	1.66E-06	Altered group
36	PTRHD1	2p23.3	Amp	8 (23.53%)	>10	2.81E-09	1.66E-06	Altered group
37	RN7SKP27	2p24.1	Amp	8 (23.53%)	>10	2.81E-09	1.66E-06	Altered group
38	RN7SL117P	2p24.1	Amp	8 (23.53%)	>10	2.81E-09	1.66E-06	Altered group

**39**	RN7SL610P	2p23.3	Amp	8 (23.53%)	>10	2.81E-09	1.66E-06	Altered group
**40**	RNA5SP87	2p24.1	Amp	8 (23.53%)	>10	2.81E-09	1.66E-06	Altered group
